# A Theoretical Analysis of Relations between Pressure Changes along Xylem Vessels and Propagation of Variation Potential in Higher Plants

**DOI:** 10.3390/plants10020372

**Published:** 2021-02-15

**Authors:** Ekaterina Sukhova, Elena Akinchits, Sergey V. Gudkov, Roman Y. Pishchalnikov, Vladimir Vodeneev, Vladimir Sukhov

**Affiliations:** 1 Department of Biophysics, N.I. Lobachevsky State University of Nizhny Novgorod, 603950 Nizhny Novgorod, Russia; n.catherine@inbox.ru (E.S.); akinchits_elena@inbox.ru (E.A.); s_makariy@rambler.ru (S.V.G.); v.vodeneev@mail.ru (V.V.); 2 Prokhorov General Physics Institute of the Russian Academy of Sciences, 119991 Moscow, Russia; rpishchal@kapella.gpi.ru

**Keywords:** variation potential, modeling of signal propagation, increased hydraulic pressure, xylem vessel, higher plants

## Abstract

Variation potential (VP) is an important long-distance electrical signal in higher plants that is induced by local damages, influences numerous physiological processes, and participates in plant adaptation to stressors. The transmission of increased hydraulic pressure through xylem vessels is the probable mechanism of VP propagation in plants; however, the rates of the pressure transmission and VP propagation can strongly vary. We analyzed this problem on the basis of a simple mathematical model of the pressure distribution along a xylem vessel, which was approximated by a tube with a pressure gradient. It is assumed that the VP is initiated if the integral over pressure is more than a threshold one, taking into account that the pressure is transiently increased in the initial point of the tube and is kept constant in the terminal point. It was shown that this simple model can well describe the parameters of VP propagation in higher plants, including the increase in time before VP initiation and the decrease in the rate of VP propagation with an increase in the distance from the zone of damage. Considering three types of the pressure dynamics, our model predicts that the velocity of VP propagation can be stimulated by an increase in the length of a plant shoot and also depends on pressure dynamics in the damaged zone. Our results theoretically support the hypothesis about the impact of pressure variations in xylem vessels on VP propagation.

## 1. Introduction

Electrical signals are important long-distance signals that are likely to participate in the induction of a systemic response in plants after the local action of stressors [1]. Action potential, system potential, and variation potential (VP) are considered to be key electrical signals in higher plants [1,2,3,4,5,6,7,8]. It is known that action potential is a short-term impulse signal (depolarization followed by repolarization) that is induced by non-damage stressors; it is related to the transient activation of ion channels (Ca^2+^, K^+^, and anion channels) and inactivation of H^+^-ATPase in the plasma membrane [7]. The system potential is a hyperpolarization signal that is likely to be related to the transient activation of the H^+^-ATPase [9,10]. The VP is a long-term electrical signal that is induced by local damages (burning, heating, crush, etc.) [5]. It is mainly related to the activation of Ca^2+^ channels and the inactivation of H^+^-ATPase [3,5,11]; however, the transient activation of other anion channels can also participate in VP generation [5,7]. 

It is known that electrical signals are likely to influence numerous physiological processes in plants [1,2,3,4], including activation of the expression of defense genes [12,13,14] and production of stress phytohormones [14,15,16,17,18]; increase in the respiratory rate [19,20,21] and ATP content of leaves [22]; changes in transpiration [23,24,25], water content [26], and photosynthetic processes [27,28,29,30,31,32]; and suppression of phloem mass flow [33,34] and plant growth [35]. There are several arguments supporting the participation of electrical signals in the induction of physiological changes after local actions of stressors. (i) Propagation of electrical signals through some parts of the plant is necessary for the induction of changes in the expression of defense genes [14], respiration [19,22], photosynthesis [36], ATP content [22], and the synthesis of jasmonic acid [14] in the parts [14,19,22]. (ii) Magnitudes of electrical signals are significantly related to magnitudes of photosynthetic changes [30]. (iii) The earliest photosynthetic changes are observed near conductive bundles [23,36], which are the main channel of propagation of electrical signals. 

Increase in plant tolerance to the action of stressors is probably the main result of these physiological responses [1,6,37,38]; in particular, electrical signals (including VP) can modify the heat tolerance of photosynthetic machinery [38,39,40,41,42]. The increased plant tolerance to actions of stressors can be potentially related to the propagation of both electrical signals and slow specific signals; at least, the effect is shown under the action of excessive light [43].

Thus, propagation of electrical signals (especially VP, which is caused by the action of dangerous damages) is expected to play an important role in the adaptation of a plant organism to the action of stressors; modification of this propagation can be considered as a potential way of management of plant tolerance to adverse factors. However, mechanisms of VP propagation are still under discussion [5]. VP is considered to be a local electrical response to the propagation of non-electrical signals [3,5]. It is supported by a decrease in VP amplitude and propagation rate with an increase in the distance from the damaged zone and passing of the variation potential through inactive or dead tissues [1]. Chemical signals, which are based on the transmission of a specific wound substance from the zone of damage to other parts of the plant through xylem vessels, and hydraulic signals, which are based on the propagation of pressure increase from the zone of damage to other parts of the plant through these vessels, are the most probable hypothetical mechanisms of VP propagation [1,5,44,45,46,47]. Initiation of a chemical signal can be related to its fast production or release from an internal source in the damaged zone after the action of the stressor [5]. Pressure increase in the damaged zone (the hydraulic signal) can be related to both (i) direct pressure increase in xylem vessels and apoplast under burning, heating, or mechanical wounding [47] and (ii) indirect increase caused by the release of osmotically active chemicals (ions, sugars, etc.) from destructed cells. The last mechanism is supported by a long-term increase in the plant stem diameter (several minutes), which is observed after short-term burning and shows the pressure increase [45].

Potentially, both signals activate Ca^2+^ channels, which can be mechanosensitive (for hydraulic signals) or ligand dependent (for chemical signals) [3,4,5,6,7,8,48]; however, direct inactivation of H^+^-ATPase is also considered [47]. Other mechanisms of VP induction also cannot be excluded; e.g., changes in pressure can influence the rate of water mass flow in xylem vessels [45]. In turn, the changes in the water mass flow rate can potentially modify a streaming potential (e.g., see [49,50,51] for trees). However, changes in the electrical potential lag behind the changes in the water mass flow rate (about 1–2 h and more [50,51]); thus, participation of the streaming potential in VP propagation does not seem to be very probable, because VP propagation usually requires tens of seconds or a few minutes [46]. 

Both chemical and hydraulic hypotheses have some limitations. A chemical signal is too slow because the rate of diffusion of chemical agents in a plant is less than about 1 mm s^−1^ [52]; in contrast, the rate of VP propagation is from 0.2 to 20 mm s^−1^ [1]. Additionally, the wound substance is still not revealed; H_2_O_2_, systemin, jasmonic and abscisic acids, glutamate, and other agents have been discussed to be potential wound substances [5,53,54,55,56,57,58,59]. In contrast, a hydraulic signal is too fast because the rate of propagation of increased pressure is at least 10 cm s^−1^ [60] or more [5,61]. 

There are combined hypotheses that are based on the accelerated transmission of the wound substance by changes in hydraulic pressure: hydraulic dispersion [45] and turbulent diffusion [62,63]. These combined hypotheses can potentially explain the propagation of a wound substance for 10–15 cm [62,63,64]; however, it is shown [46] that VP can propagate for at least 45 cm, with a moderate decrease in the propagation rate and amplitude in grape plants. A high VP propagation rate a long distance from the damaged zone (e.g., 14 mm s^−1^ at 15 cm from this zone in pea seedlings [65]) is not in accordance with the combined hypotheses, too. 

As a result, a hydraulic hypothesis of VP propagation seems to be more perceptive than others and requires further analysis, especially for plants with long shoots (tens of centimeters) and a high rate of VP propagation (tens of millimeters per second and more). Our previous works [62,63,64] have shown that mathematical modeling is an effective tool for theoretical analysis of mechanisms of VP propagation in higher plants. Thus, the aim of the current work was the development of a simple model of hydraulic propagation of variation potential and theoretical analysis of relations between pressure changes along xylem vessels and VP propagation in higher plants on the basis of this model. 

## 2. Model of Hydraulic Propagation of Variation Potential 

**Figure 1:**
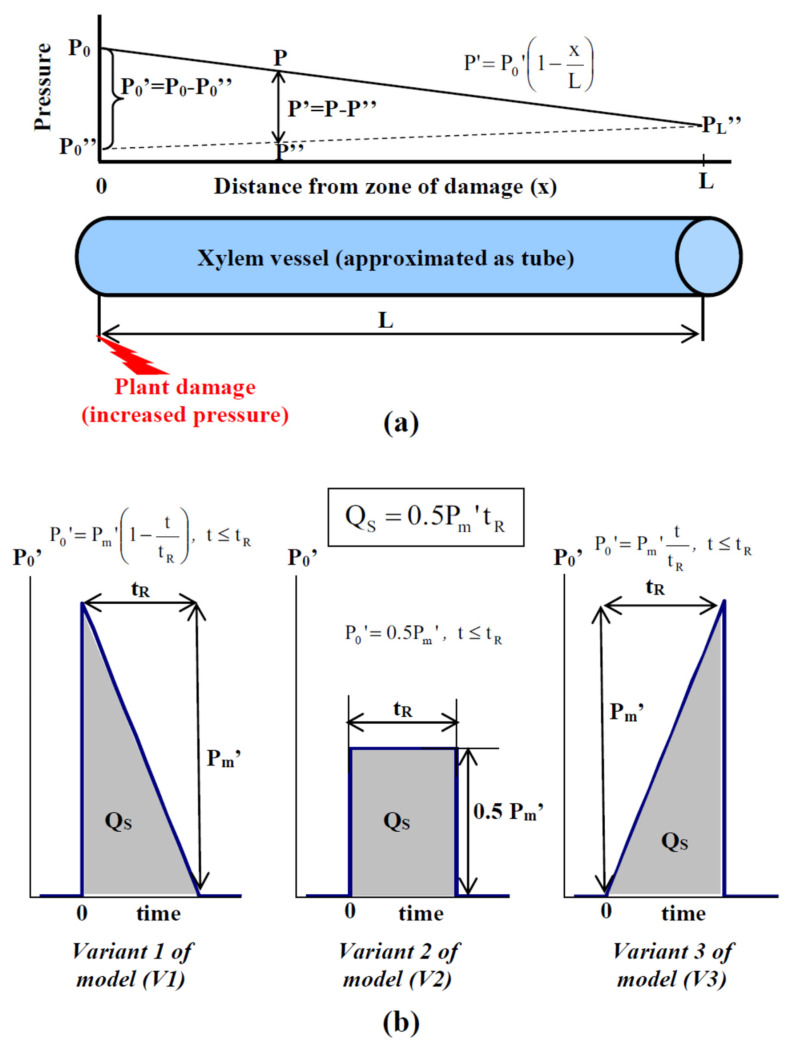
**(a)** Scheme of the pressure distribution in a xylem vessel, which is approximated by a tube of length equaling to L. P_0_”, P_L_”, and P” are pressures without damage in the initial (x = 0), terminal (x = L), and intermediate (0 < x < L) points of the vessel, respectively; it is assumed that P_0_”, P_L_”, and P” are not dependent on time; P” is a linear function of x (see Equation (3) in Section 2). P_0_ and P are pressures in the initial (x = 0) and intermediate (0 < x < L) points of the vessel, respectively, after the start of action of a stressor; they are time dependent, and P is also dependent on x. P_0_’ and P’ are the increases in pressure above pressure values without stimulation. It is also assumed that a stimulation acts on the initial point of the vessel and induces the transient increase in P_0_’ and, therefore, P’. The equation that describes the relations between P_0_’ and P’ is also shown in the figure (see Section 2 for details). **(b)** Variants of stimulation-induced increases in pressure (P_0_’) that are used in our analysis. An instant initial increase in pressure and a subsequent linear decrease during the time of response (t_R_) are assumed for Variant 1 (V1) of the variation potential (VP) propagation model. V1 is in accordance with the short-term and highly intensive action of damages (e.g., burning or crush, which are widely used damages for VP induction [1,5]). Constant increased pressure during the time of response is assumed for Variant 2 (V2) of the model. V2 is in accordance with the long-term and constant action of a stressor (e.g., an artificial increase in pressure in a part of the shoot, which was used in some experiments [44]). The long-term increase in pressure during t_R_ and its instant decrease after that are assumed for Variant 3 (V3) of the model. V3 most probably is in accordance with the increase in the intensity of the action of a stressor (e.g., gradual heating, which can be also used for the induction of VP [63,69]). The maximum pressure increases are P_m_’ for V1 and V3 and 0.5P_m_’ for V2. Q_S_ is the integral of total changes in pressure (marked gray) in the initial point of the xylem vessel (integral of P_0_’). The equation for Q_S_ is shown in the figure (it is similar for V1, V2, and V3). Equations that describe the dependence of P_0_’ on time are also shown in the figure (see Section 2 for details).

We use the simplest description of a xylem vessel as a tube (in accordance with [45,61,66]) with the length (L) and a constant radius (Figure 1a). It should be noted that the description does not include perforated plates, which can be observed in the vessel [66], because the total thicknesses of the plates are much less than the total length of the vessel, i.e., the pressure decrease in each plate should be very small and can be excluded from the analysis. Additionally, we do not consider decreasing the vessel radius with increasing distance from the basal part of the plant shoot [67] in the model. Potentially, the effect can modify the pressure gradient along the xylem vessel. However, the radius changes are mainly observed at long distances (meters) in trees [67]; in contrast, we analyze distances that are no more than 0.5 m in our work. 

Equation (1) could be derived on the basis of the Poiseuille law [68] for a tube with a constant radius:

(1) $$\frac{dP}{dx}=\frac{P_{0}-P_{L}\prime \prime }{L}$$

where P is the stationary pressure at x, x is the distance from the initial point of the tube, P_0_ is the pressure in the initial point of the tube, P_L_” is the pressure in the terminal point of the tube, and L is the length of the tube. For the solution of Equation (1), we have:

(2) $$P=P_{0}-\frac{P_{0}-P_{L}\prime \prime }{L}x$$

Next, we assume that the pressure value consists of two components: the pressure without damage (P”) and its increase under damage (P’). It is also assumed that the damage increases the pressure above the values of pressure without the stimulation in the initial (x = 0) point of the vessel (P_0_’), while the increase in pressure in the terminal point of the tube (x = L) is absent (the pressure is constant in the point). In this case, Equation (2) is transformed into Equations (3) and (4):

(3) $$P\prime \prime =P_{0}\prime \prime -\frac{P_{0}\prime \prime -P_{L}\prime \prime }{L}x$$

(4) $$P\prime =P_{0}\prime \left(1-\frac{x}{L}\right)$$

where P_0_” is the pressure without damage in the initial (x = 0) point of the vessel. 

We consider three variants of the P_0_’ dynamics in the xylem vessel under damage (Figure 1b). An instant initial increase in pressure (for t = 0) and the subsequent linear decrease during the time of response (t_R_) are assumed for Variant 1 (V1) of the variation potential (VP) propagation model. V1 is in accordance with short-term and highly intensive action of damages (e.g., burning or crush, which are widely used damages for VP induction [1,5]). Equation (5) is used for describing P_0_’ dynamics in V1 for t ≤ t_R_; P_0_’ is 0 for t > t_R_.

(5) $$P_{0}\prime =P_{m}\prime \left(1-\frac{t}{t_{R}}\right)$$

where P_m_’ is the maximum pressure increase in the initial point of the vessel. 

Constantly increased pressure during the time of response was assumed for Variant 2 (V2) of the model. V2 is in accordance with the long-term and constant action of a stressor (e.g., an artificial increase in pressure in a part of the shoot, which was used in some experiments [44]). Equation (6) is used for describing P_0_’ dynamics in V2 for t ≤ t_R_; P_0_’ is 0 for t > t_R_.

(6) $$P_{0}\prime =0{.5P}_{m}\prime$$

where 0.5P_m_’ is the value of a constant pressure increase in the initial point of the vessel. 

The long-term increase in pressure for t_R_ and its instant decrease after that are assumed for Variant 3 (V3) of the model. V3 most probably is in accordance with the increase in the intensity of the action of the stressor (e.g., gradual heating, which can be also used for the induction of VP [63,69]). Equation (7) is used for describing P_0_’ dynamics in V3 for t ≤ t_R_; P_0_’ is 0 for t > t_R_.

(7) $$P_{0}\prime =P_{m}\prime \frac{t}{t_{R}}$$

Q_S_ (Figure 1b) is the integral of total changes in pressure in the initial point of the xylem vessel (integral of P_0_’); Equation (8) is used for the calculation of Q_S_ in all variants of the model.

(8) $$Q_{S}=0{.5P}_{m}{\prime t}_{R}$$

It is assumed that the induction of VP (or detection of this induction) requires that the integral of P’ in some point of the xylem vessel be more than the threshold value (Q_th_), i.e., $P_{m}\prime \left(1-\frac{x}{L}\right)\int_{0}^{t}f\left(t\right)\mathrm{dt}\geq Q_{\mathrm{th}}$, where f(t) is Equation (5), (6), or (7) for V1, V2, or V3, respectively. As a result, the time of initiation of VP (t_th_) could be derived from Equation (9):

(9) $$P_{m}\prime \left(1-\frac{x}{L}\right)\int_{0}^{t_{\mathrm{th}}}f\left(t\right)\mathrm{dt}=Q_{\mathrm{th}}$$

If Q_th_ was described as NQ_S_ (where N = Q_th_/Q_S_) or as 0.5NP_m_’t_R_ (in accordance with Equation (8)), then t_th_ could be derived from Equation (10):

(10) $$\frac{2}{t_{R}}\left(1-\frac{x}{L}\right)\int_{0}^{t_{\mathrm{th}}}f\left(t\right)\mathrm{dt}=N$$

Using Equations (5)–(7), we derived Equations (11)–(13) for describing the dependence of t_th_ on distance from the damaged zone (x) in V1, V2, and V3, respectively.

(11) $$t_{\mathrm{th}}=t_{R}\left(1-{\left(1-\frac{N}{1-x/L}\right)}^{1/2}\right)$$

(12) $$t_{\mathrm{th}}=t_{R}\frac{N}{1-x/L}$$

(13) $$t_{\mathrm{th}}=t_{R}{\left(\frac{N}{1-x/L}\right)}^{1/2}$$

It is important that Equations (11)–(13) be usable at x ≤ x_max_, where x_max_ is the maximal distance for VP propagation. x_max_ is calculated on the basis of Equation (14), which was derived from Equations (4) and (8):

(14) $$Q_{S}\left(1-x_{\max}/L\right)={\mathrm{NQ}}_{S}$$

Equation (14) was transformed to Equation (15):

(15) $$x_{\max}=\left(1-N\right)L$$

Using Equations (11)–(13), we derived Equations (16)–(18) for describing the dependence of the rate of VP propagation (V_VP_) on the distance from the damaged zone (x) in V1, V2, and V3, respectively. These equations were based on a description of V_VP_ as dx/dt_th_. Equations (16)–(18) can be used at x ≤ x_max_.

(16) $$V_{\mathrm{VP}}=\frac{2L}{t_{R}N}{\left(1-\frac{N}{1-x/L}\right)}^{1/2}{\left(1-x/L\right)}^{2}$$

(17) $$V_{\mathrm{VP}}=\frac{L}{t_{R}N}{\left(1-x/L\right)}^{2}$$

(18) $$V_{\mathrm{VP}}=\frac{2L}{t_{R}}{\left(1-x/L\right)}^{3/2}N^{-1/2}$$

Thus, Equations (11)–(13) can be used for theoretical analysis of the dependence of the time of initiation of VP on the distance from the damaged zone in different variants of the hydraulic model of VP propagation. Equations (16)–(18) can be used for theoretical analysis of the dependence of the rate of VP propagation on the distance from the damaged zone in different variants of the hydraulic model of VP propagation.

## 3. Results

### 3.1. Verification of the Developed Model of Hydraulic Propagation of Variation Potential

The developed model of hydraulic propagation of variation potential was based on a description of the stationary pressure distribution along a tube that imitated a xylem vessel; on a description of dynamics of pressure changes in the initial point of this tube (damaged zone), including three variants of the dynamics; and on the assumption of the initiation of VP after the integral of pressure reached the threshold value (see Figure 1 and Section 2 for details). The first question of theoretical analysis was, Could our simple model imitate experimental results well?

Our model was based on three parameters: the length of the xylem vessel (L), the duration of hydraulic changes in the damaged zone (t_R_), and the ratio (N) of the integral of pressure increase (P’), which was necessary for induction of variation potential, to the total integral of the pressure increase in the zone of damage (P_0_’). L was estimated on the basis of the length of the plant shoot in each used experimental work, and t_R_ and N were assumed for each experimental variant on the basis of the least-squares method. It should be noted that t_R_ and N should be strongly dependent on parameters of damage; increase in the intensity of damage should decrease N and could increase t_R_, and an increase in the duration of damage should increase t_R_ and could decrease N; i.e., t_R_ and N could be strongly varied in different experiments. We used Variant 1 (V1) of the model for simulating the action of short-term damages (burning or crush) and Variant 2 (V2) for simulating a long-term artificial increase in pressure. 

**Figure 2:**
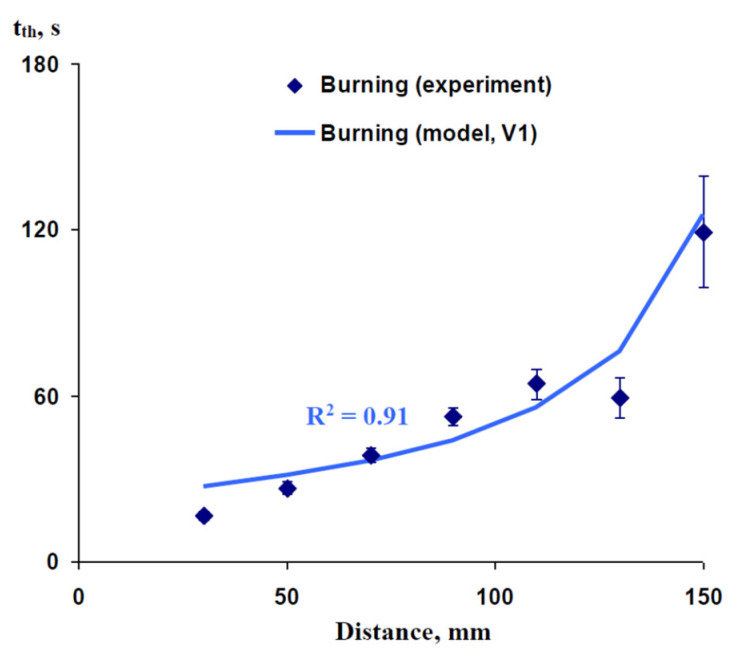
Experimental and simulated dependence of the time of initiation of variation potential (*t*_th_) on the distance from the burned zone in wheat seedlings. We used experimental data from our previous work [62]. The length of the xylem vessel (L) is assumed as 200 mm (on the basis of [62]). The duration of hydraulic changes in the damaged zone (t_R_) is assumed as 200 s. The ratio of the P’ integral, which is necessary for variation potential induction, to the total P_0_’ integral in the zone of damage (N) is assumed as 0.13. Only Variant 1 (V1) of the model was used in this analysis (see Figure 1b). It is assumed that V1 (instant initial increase in pressure and subsequent linear decrease) is in accordance with the action of short-term burning. R^2^ is the determination coefficient.

Figure 2 shows the experimental (wheat seedlings, data from work [62]) and simulated dependence of the time of initiation of variation potential (t_th_) on the distance from the burned zone. In the experiment, this time non-linearly increased with an increase in the distance from the zone of damage; t_th_ was about 110 s at a 150 mm distance. The simulated dependence was very similar to the experimental one (the determination coefficient (R^2^) between these dependences was 0.91); i.e., the developed model could effectively describe the dependence of t_th_ on distance in burning-induced VP propagation in wheat seedlings. It is important that the assumed t_R_ was 200 s, because the duration of pressure changes in the work [62] was at least 200 s (see Figure 3 in [62]). It showed that the experimental dependence could be imitated by the model in a realistic duration of pressure increase in the zone of damage.

**Figure 3:**
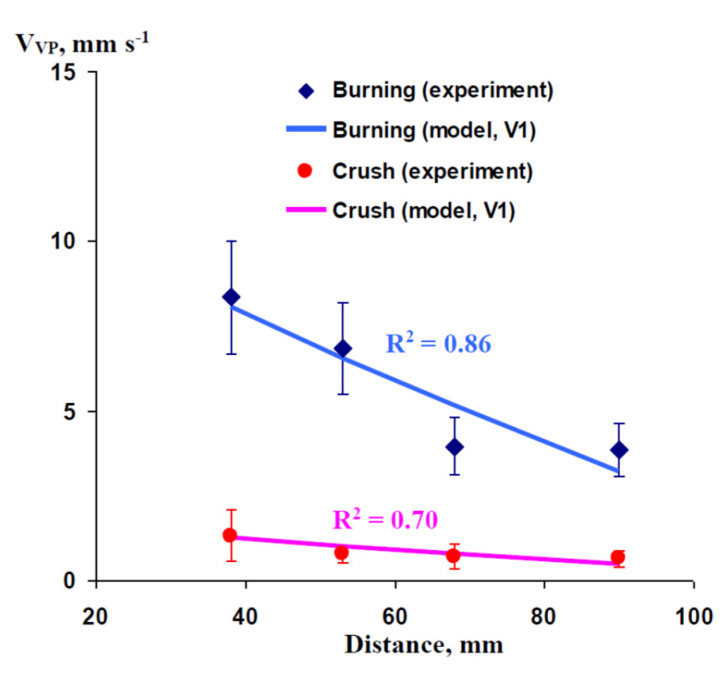
Experimental and simulated dependence of the rates of propagation of variation potential (V_VP_) on the distance from the damaged zone in pea seedlings. We used experimental data from our previous work [63], which analyzed the propagation of variation potential for 275 mm. The duration of hydraulic changes in the damaged zone (t_R_) is assumed as 50 s (for burning) or 325 s (for crush). The ratio of the P’ integral, which is necessary for variation potential induction, to the total P_0_’ integral in the zone of damage (N) is assumed as 0.58 (for both burning and crush). Only Variant 1 of the model (V1) of the model was used in this analysis (see Figure 1b). It is assumed that V1 (instant initial increase in pressure and subsequent linear decrease) is in accordance with the action of short-term burning or mechanical wounding. R^2^ is the determination coefficient.

Figure 3 shows the experimental (pea seedlings, data from work [63]) and simulated dependence of the propagation rate of VP (V_VP_) on the distance from the damaged zone. Two types of short-term damages were used in this analysis: burning of leaf and crushing of leaf. The propagation rate of burning-induced VP was about 8.4 mm s^−1^ at a 38 mm distance from the zone of damage; V_VP_ strongly decreased with an increase in the distance from the burned zone and was about 3.9 mm s^−1^ at a 90 mm distance. The model well simulated the experimental dependence (R^2^ was 0.86) at t_R_ = 50 s and N = 0.58.

The propagation rate of crush-induced VP had a similar dependence on the distance from the damaged zone; however, V_VP_ was significantly lower (V_VP_ = 1.3 mm s^−1^ at 38 mm and V_VP_ = 0.6 mm s^−1^ at 90 mm). The developed model well simulated this dependence at the same N (N = 0.58) and t_R_ equaling 325 s. It should be noted that the integral of total pressure changes (Q_S_) was also constant at constant N because N ≈ (Q_S_)^−1^ = (0.5P_m_’t_R_)^−1^, where P_m_’ is the maximal pressure in the damaged zone (see Figure 1 and Section 2); i.e., the increase in t_R_ corresponded to a proportional decrease in P_m_’. In contrast, the dependence of the propagation rate of crush-induced VP on the distance from the damaged zone could not be simulated only by changes in N (without changes in t_R_); i.e., an increase in the duration of pressure changes was necessary for an adequate description of experimental results at crush (data not shown).

**Figure 4:**
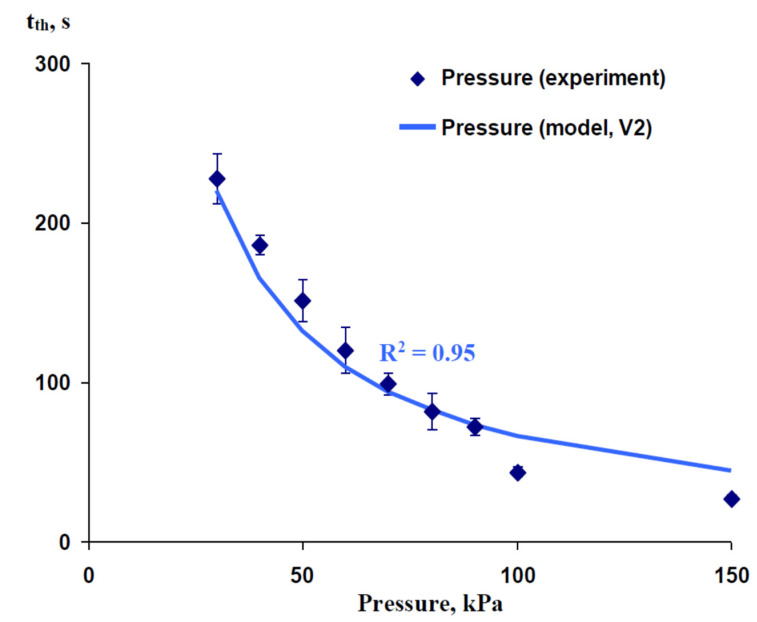
Experimental and simulated dependence of the time of initiation of variation potential (t_th_) on the pressure dynamics acting on the tip of the stem of pea seedlings. The experimental t_th_ is calculated (means and standard errors) on the basis of the results that were presented in [44]; only a 60 mm distance from the zone of pressure action is analyzed. Only Variant 2 (V2) of the model was used in this analysis (see Figure 1b). It is assumed that V2 (constant increased pressure for all time of response) is in accordance with the artificial action of the increased pressure in [44]. The length of the xylem vessel (L) is assumed as 120 mm (on the basis of [44]). The duration of pressure increase (t_R_) is assumed as 1000 s because this duration was more than any experimental *t*_th_ and is in accordance with the artificial long-term increase in pressure. The ratio of the P’ integral, which is necessary for variation potential induction, to the total P_0_’ integral in the zone of damage (N) is assumed as 0.11 for 30 kPa (N_30_). Under other values of current pressure, we used N, which were calculated as $N_{30}\frac{30}{current\;pressure}$ because the total integral of pressure in the zone of damage was proportional to the current pressure. R^2^ is the determination coefficient.

Figure 4 shows that the action of increased pressure on roots and the lower part of the shoot induced VP in a pea stem in the experiment. Experimental points were calculated on the basis of Figure 8 in [44]; only a 60 mm distance from the zone of pressure action was analyzed. t_th_ decreased with an increase in pressure; the dependence was not linear. For simulation of this dependence, we used V2 (constant increase in pressure); t_R_ was assumed as 1000 s because this time interval was more than any t_th_ in the experiment and was in accordance with the long-term action of increased pressure on the plant. N was assumed as 0.11 for 30 kPa (N_30_). Other N values, which corresponded to other current pressures, where calculated as $N_{30}\frac{30}{current\;pressure}$ because N is proportional to (Q_S_)^−1^ = (0.5P_m_’t_R_)^−1^. It was shown that the simulated dependence was in good accordance with the experimental one (R^2^ = 0.95); small differences between experimental t_th_ and simulated ones were only observed at high pressures.

Thus, results of this part of the analysis showed that the developed simple model effectively described some experimental results, including the dependence of t_R_ and V_VP_ on the distance from the damaged zone (initial point of the simulated xylem vessel) and dependence of t_R_ on the value of the increased pressure, which acted on the initial point; i.e., this model could be used for further analysis of the relations between the distribution of pressure changes along the xylem vessel and the propagation of variation potential. 

### 3.2. Theoretical Analysis of the Influence of the Length of a Xylem Vessel and Pressure Dynamics in Its Initial Point on the Propagation of Variation Potential

In the next stage of investigation, we analyzed the influence of the length of the xylem vessel and pressure dynamics in its initial point on the propagation of variation potential using the developed model. We used t_R_ = 50 s and N = 0.3 for qualitatively revealing the influence of L and the shape of pressure dynamics on VP propagation. 

**Figure 5:**
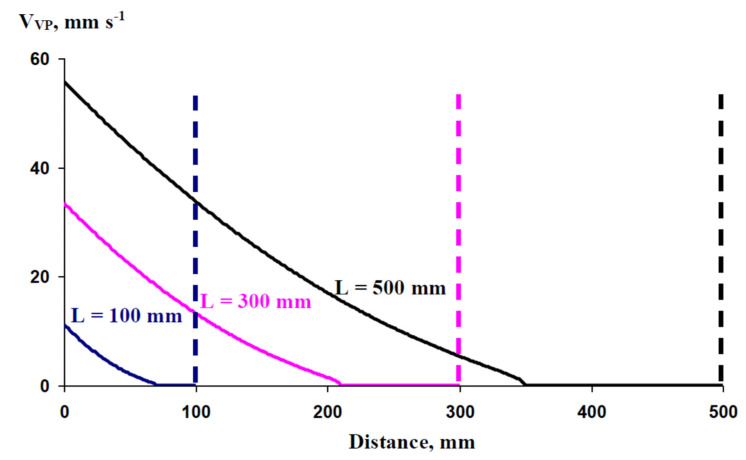
Simulated dependence of the propagation rates of variation potential (V_VP_) on the distance from the damaged zone at different lengths of the xylem vessel (L). L is 100, 300, and 500 mm. The duration of hydraulic changes in the damaged zone (t_R_) is assumed as 50 s. The ratio of the P’ integral, which is necessary for variation potential induction, to the total P_0_’ integral in the zone of damage (N) is assumed as 0.3. Variant 1 (V1) of the model was used in this analysis (see Figure 1b). Dotted lines show the lengths of the vessels (L) that were used for analysis.

Figure 5 shows that the dependence of V_VP_ on the distance from the damaged zone, which was simulated by the model (V1), was modified by the length of the xylem vessel, which could be approximately considered as the length of the plant shoot. It was shown that an increase in L was accompanied by an increase in the propagation rate of VP, a decrease in the decrement of V_VP_ with an increase in the distance from the damaged zone, and an increase in the maximal distance of VP propagation (x_max_). Thus, the analysis of the model theoretically showed that a hydraulic signal could be propagated in plants with different lengths of xylem vessels (i.e., with different lengths of shoots); moreover, this propagation was likely to be stimulated by an increase in the xylem vessel length. 

**Figure 6:**
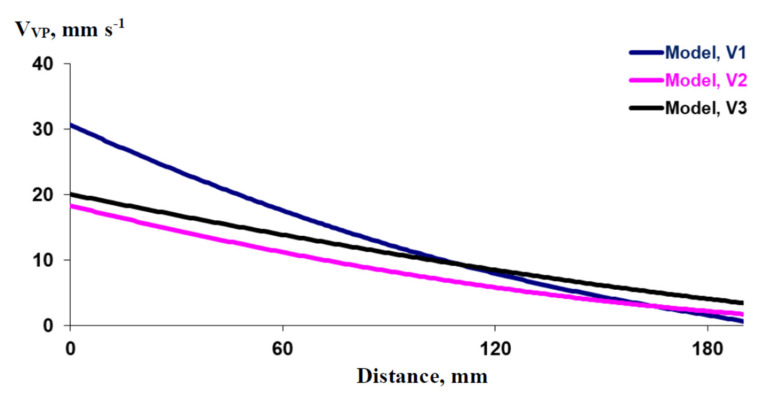
Simulated dependence of the propagation rates of variation potentials (V_VP_) on the distance from the damaged zone in different variants of the model (see Figure 1b). The length of the xylem vessel (L) is assumed as 275 mm. The duration of hydraulic changes in the damaged zone (t_R_) is assumed as 50 s. The ratio of the P’ integral, which is necessary for variation potential induction, to the total P_0_’ integral in the zone of damage (N) is assumed as 0.3. Variant 1 (V1) of the model (instant initial increase in pressure and subsequent linear decrease), Variant 2 (V2) of the model (constant increased pressure for all time of response), and Variant 3 (V3) of the model (long-term increase in pressure and subsequent instant decrease in it) were used.

Figure 6 shows that the shape of the dynamics of pressure in the damaged zone could influence VP propagation: changes in this shape modified the dependence of V_VP_ on the distance from the zone of stimulation. It was shown that there were strong differences in the decrement of V_VP_ with an increase in the distance from the initial point on using different variants of the model. The maximal decrement was observed on using V1 (V_VP_ = 30.6 mm s^−1^ at x = 0 mm and V_VP_ = 1.6 mm s^−1^ at x = 180 mm; i.e., the decrease in the rate of VP propagation was 95%), a moderate decrement was observed on using V2 (V_VP_ = 18.3 mm s^−1^ at x = 0 mm and V_VP_ = 2.2 mm s^−1^ at x = 180 mm; i.e., the decrease in the rate of VP propagation was 88%), and the minimal decrement was observed on using V3 (V_VP_ = 20.1 mm s^−1^ at x = 0 mm and V_VP_ = 4.1 mm s^−1^ at x = 180 mm; i.e., the decrease in the rate of VP propagation was 80%). It should be additionally noted that V_VP_ at long distances from the damaged zone was maximal on using V3 and minimal on using V1; i.e., the propagation rates were inverted in comparison to the propagation rates at small distances. Thus, the results theoretically showed that the pressure dynamics in the damaged zone, which could be strongly related to the characteristics of the action of the stressor (in particular, to the dynamics of its action), were an additional potential factor in the regulation of VP propagation through the plant body.

## 4. Discussion

Variation potential is an important electrical signal in plants that is generated after the local action of dangerous stressors, regulates numerous physiological processes, and is likely to participate in plant adaptation to the action of stressors [1,2,3,4,5,6,7,8]. Revealing mechanisms of VP propagation can be the basis for the development of additional approaches for the regulation of this signal and, probably, modification of the plant’s tolerance to stressors. A hydraulic hypothesis of VP propagation supposes that the variation potential is a local electrical response to the propagation of increased pressure along xylem vessels after local damage to plants [2,4,5,47]; this propagation induces the activation of mechanosensitive ion channels (probably Ca^2+^ channels), stimulates Ca^2+^ influx, and, thereby, inactivates H^+^-ATPase in the plasma membrane. However, the rate of transmission of the increased pressure along xylem vessels probably is much larger than the rate of VP propagation (at least 10 cm s^−1^ [60] or more [5,61] for a hydraulic wave and 0.2 to 20 mm s^−1^ for VP [1]); as a result, this problem requires further analysis.

In the current work, we theoretically analyze relations between pressure changes along xylem vessels and the propagation of variation potentials in higher plants. Our analysis supports the participation of hydraulic signals in VP propagation and theoretically shows some properties of this participation. The following important points are shown in our work. 

A simple model of the hydraulic propagation of VP is developed in our work. The model is based on the description of the stationary pressure distribution along a tube that imitates a xylem vessel; on the description of dynamics of pressure changes in the damaged zone (the initial point of the tube), including three variants of the dynamics; and on the assumption of the initiation of VP after the integral of pressure reaches the threshold value. Analytical solutions to the equations describing t_th_, V_VP_, and x_max_ are obtained for all variants of the model. There are only three parameters (L, t_R_, and N) that are necessary for this model. 

The proposed model well describes the number of experimental results (from works [44,62,63]), including the dependence of t_th_ and V_VP_ on the distance from the damaged zone and the dependence of t_th_ on the value of the increased pressure acting on the initial point of the xylem vessel (see Figure 2, Figure 3 and Figure 4). It is important that the assumed values of model parameters (L, t_R_, and N) be in the physiological range, and additional assumptions are not necessary. In contrast, our previous VP models, which are based on the propagation of chemical signals, require additional assumptions, in particular an extremely high diffusion coefficient for the wound substance (D = 0.06–0.012 cm^2^ s^−1^) [62,63,64]. This high value of D is hypothesized to be related to the non-laminar water flow in the xylem and the induction of turbulent diffusion [5,62]. However, it is unlikely that turbulent diffusion can transmit a wound substance by tens of centimeters (we simulated the propagation of the wound substance and VP for 10–15 cm using our previous models [62,63,64]; the radioactive marker was transmitted for less than 15 cm for 200 s [62]); in contrast, VP can propagate for 28 [65]-40 cm [46] and more.

It should be additionally noted that the model (especially Variant 1) can describe both linear (e.g., see Figure 3) and non-linear (e.g., Figure 5 and Figure 6) dependence of the rate of VP propagation on the distance from the damaged zone. The linear dependence is observed at high N (e.g., N = 0.58; Figure 3), and the non-linear dependence is found at low N (e.g., N = 0.3; Figure 5 and Figure 6). The influence of L and t_R_ on the linearity of the dependence is rather weak. Both linear and non-linear dependence can be observed in experiments; e.g., investigation of VP propagation in wheat seedlings [69] shows that non-linear dependence is observed in control plants and linear dependence is observed in plants treated by chronic β-radiation. It is probable that the differences can be caused by differences in N in these cases. 

Theoretical analysis shows that VP propagation can be stimulated by an increase in the length of the xylem vessel (increase in the rate of VP propagation, decrease in the V_VP_ decrement with an increase in the distance from the damaged zone, increase in x_max_). It seems to be unusual and requires further verification; however, it can explain VP propagation over long distances (tens of centimeters [15,46,65]) and experimentally detected high rates of VP propagation over such distances (e.g., 20 mm s^−1^ at a 17–20 cm distance from the burned zone [15]).

Theoretical analysis also shows that the dynamics of pressure changes in the damaged zone can strongly influence VP propagation; in particular, a decrement in V_VP_ with an increase in the distance from the zone of stimulation was maximal on using V1 (the instant initial increase in pressure and the subsequent linear decrease) and minimal on using V3 (long-term increase in pressure and subsequent instant decrease in it). It is interesting that our previous work [63] showed that the decrement in the propagation rate of variation potential is maximal after induction of VP in pea seedlings by leaf burning (short-term action of a stressor with high intensity, which is similar to V1) and it is practically absent after induction of VP by gradual heating to 60 °C for about 4 min (long-term and slow increase of intensity of stressor action, which is similar to V3). It should be also noted that V_VP_, which was calculated on the basis of data from [62], was about 2 mm s^−1^ at about 4 cm from the burning zone (2 s burning) in wheat seedlings; in contrast, V_VP_ was 0.85 mm s^−1^ at about 4 cm from the heated zone (gradual heating) in similar seedlings [69]. The results, which are in qualitative accordance with Figure 6 (V1 for burning and V3 for heating), additionally show that different types of damage can induce different rates of VP propagation. It cannot be excluded that the relation between the dynamics of pressure changes in the damaged zone and the parameters of VP propagation can be the basis of transmission of information about the type of stimulus to undamaged parts of a plant; the possibility of this transmission by VP is actively discussed now [1].

On the whole, our model can be considered as the first step in the simulation of the hydraulic propagation of VP, because the current mathematical models [7] of variation potentials describe propagation of a wound substance through turbulent diffusion [62,63,64] and hydraulic dispersion [70,71]. The results of its analysis seem to be encouraging; however, there are some limitations, which require further development of the model. 

(i) The single tube is a very simple description of xylem vessels. It does not consider the complicated connections between xylem vessels in plants [65,72,73] or the decreasing vessel radius with increasing distance from the basal part of the plant shoot [67]. Potentially, the last point can be important for simulation of VP propagation in trees (on distances equaling several meters). 

(ii) The model is based on a minimal description of the threshold for VP (as a function of integral of pressure changes) and does not simulate the amplitude of VP, which can be strongly correlated to magnitudes of physiological responses (e.g., photosynthetic response [30]). It is known that there are differences in the dependence of the VP amplitude on the distance from the damaged zone: the amplitude is not significantly dependent on the distance [30,46,63], and the amplitude significantly decreases with an increase in the distance [64,65,69]. The type of dependence can be related to the type of stressor: significant changes in the VP amplitude with an increase in the distance are absent after burning and heating but are observed after crushing of leaves in pea seedlings [63]. The differences can be explained by the saturation of the VP amplitude under high pressure values. Equations with saturation (e.g., like the equation used in our model of chemical VP propagation [63,64]) are expected to be effective in the simulation of the relation between hydraulic signals and VP amplitude. Thus, the simulation of relations is an important future task. 

(iii) The model is based on a stationary solution because it is assumed that the rate of hydraulic wave propagation is high in comparison to the VP propagation rate [5,61]. However, there are alternative approaches for analysis of the rate of increased pressure propagation that describe the propagation in animal vessels [74,75]. It is important that the approaches be able to describe the dependence of the rate of increased pressure propagation on the Young’s modulus (E) and the geometry of the vessel [75]. However, the typical propagation rates in animal vessels are several meters per second [75], significantly more than rates of VP propagation [1,5]. Moreover, the rate of increased pressure propagation is proportional to E^1/2^, and E is about 0.48 MPa in animal vessels [75] and about 3000–3500 MPa in the xylem of plants (tobacco) [76]. This means that the rate of increased pressure propagation in a plant should be way higher than that in animal vessels. Considering this point, we suppose that a description of the nonstationary increased pressure propagation is not crucial for modeling VP propagation in plants. 

(iv) Finally, this model does not include a mechanistic description of VP generation, which was developed in our earlier works [7,64]. The description can be based on complex models of plant electrogenesis [7,64,77] and can be potentially used for the prediction of relations between VP propagation, changes in the concentration of ions in the cytoplasm and apoplast, and physiological changes in plant cells. 

All these are important tasks for the future development of hydraulic models of VP propagation.

## Data Availability

The data presented in this study are available on request from the corresponding author.

## References

[1] Sukhov V., Sukhova E., Vodeneev V. (2019). Long-distance electrical signals as a link between the local action of stressors and the systemic physiological responses in higher plants. Progr. Biophys. Mol. Biol..

[2] Trebacz K., Dziubinska H., Krol E., Baluška F., Mancuso S., Volkmann D. (2006). Electrical signals in long-distance communication in plants. Communication in Plants. Neuronal Aspects of Plant Life.

[3] Fromm J., Lautner S. (2007). Electrical signals and their physiological significance in plants. Plant Cell Environ..

[4] Gallé A., Lautner S., Flexas J., Fromm J. (2015). Environmental stimuli and physiological responses: The current view on electrical signaling. Environ. Exp. Bot..

[5] Vodeneev V., Akinchits E., Sukhov V. (2015). Variation potential in higher plants: Mechanisms of generation and propagation. Plant Signal. Behav..

[6] Sukhov V. (2016). Electrical signals as mechanism of photosynthesis regulation in plants. Photosynth. Res..

[7] Sukhova E., Akinchits E., Sukhov V. (2017). Mathematical models of electrical activity in plants. J. Membr. Biol..

[8] Szechyńska-Hebda M., Lewandowska M., Karpiński S. (2017). Electrical signaling, photosynthesis and systemic acquired acclimation. Front. Physiol..

[9] Zimmermann M.R., Maischak H., Mithöfer A., Boland W., Felle H.H. (2009). System potentials, a novel electrical long-distance apoplastic signal in plants, induced by wounding. Plant. Physiol..

[10] Zimmermann M.R., Mithöfer A., Will T., Felle H.H., Furch A.C. (2016). Herbivore-triggered electrophysiological reactions: Candidates for systemic signals in higher plants and the challenge of their identification. Plant Physiol..

[11] Yudina L., Sherstneva O., Sukhova E., Grinberg M., Mysyagin S., Vodeneev V., Sukhov V. (2020). Inactivation of H+-ATPase participates in the influence of variation potential on photosynthesis and respiration in peas. Plants.

[12] Wildon D.C., Thain J.F., Minchin P.E.H., Gubb I.R., Reilly A.J., Skipper Y.D., Doherty H.M., O’Donnell P.J., Bowles D. (1992). Electrical signalling and systemic proteinase inhibitor Induction in the wounded plant. Nature.

[13] Stanković B., Davies E. (1996). Both action potentials and variation potentials induce proteinase inhibitor gene expression in tomato. FEBS Lett..

[14] Mousavi S.A., Chauvin A., Pascaud F., Kellenberger S., Farmer E.E. (2013). Glutamate receptor-like genes mediate leaf-to-leaf wound signalling. Nature.

[15] Hlavácková V., Krchnák P., Naus J., Novák O., Spundová M., Strnad M. (2006). Electrical and chemical signals involved in short-term systemic photosynthetic responses of tobacco plants to local burning. Planta.

[16] Krausko M., Perutka Z., Šebela M., Šamajová O., Šamaj J., Novák O., Pavlovič A. (2017). The role of electrical and jasmonate signalling in the recognition of captured prey in the carnivorous sundew plant *Drosera capensis*. New Phytol..

[17] Pavlovič A., Mithöfer A. (2019). Jasmonate signalling in carnivorous plants: Copycat of plant defence mechanisms. J. Exp. Bot..

[18] Farmer E.E., Gao Y.Q., Lenzoni G., Wolfender J.L., Wu Q. (2020). Wound- and mechanostimulated electrical signals control hormone responses. New Phytol..

[19] Filek M., Kościelniak J. (1997). The effect of wounding the roots by high temperature on the respiration rate of the shoot and propagation of electric signal in horse bean seedlings (*Vicia faba* L. minor). Plant Sci..

[20] Pavlovič A., Slováková L., Pandolfi C., Mancuso S. (2011). On the mechanism underlying photosynthetic limitation upon trigger hair irritation in the carnivorous plant Venus flytrap (*Dionaea muscipula* Ellis). J. Exp. Bot..

[21] Lautner S., Stummer M., Matyssek R., Fromm J., Grams T.E.E. (2014). Involvement of respiratory processes in the transient knockout of net CO_2_ uptake in *Mimosa pudica* upon heat stimulation. Plant Cell Environ..

[22] Surova L., Sherstneva O., Vodeneev V., Katicheva L., Semina M., Sukhov V. (2016). Variation potential-induced photosynthetic and respiratory changes increase ATP content in pea leaves. J. Plant Physiol..

[23] Grams T.E., Lautner S., Felle H.H., Matyssek R., Fromm J. (2009). Heat-induced electrical signals affect cytoplasmic and apoplastic pH as well as photosynthesis during propagation through the maize leaf. Plant Cell Environ..

[24] Gallé A., Lautner S., Flexas J., Ribas-Carbo M., Hanson D., Roesgen J., Fromm J. (2013). Photosynthetic responses of soybean (*Glycine max* L.) to heat-induced electrical signalling are predominantly governed by modifications of mesophyll conductance for CO_2_. Plant Cell Environ..

[25] Vuralhan-Eckert J., Lautner S., Fromm J. (2018). Effect of simultaneously induced environmental stimuli on electrical signalling and gas exchange in maize plants. J. Plant Physiol..

[26] Sukhova E., Yudina L., Gromova E., Nerush V., Vodeneev V., Sukhov V. (2020). Burning-induced electrical signals influence broadband reflectance indices and water index in pea leaves. Plant Signal. Behav..

[27] Krupenina N.A., Bulychev A.A. (2007). Action potential in a plant cell lowers the light requirement for non-photochemical energy-dependent quenching of chlorophyll fluorescence. Biochim. Biophys. Acta.

[28] Sukhov V., Sherstneva O., Surova L., Katicheva L., Vodeneev V. (2014). Proton cellular influx as a probable mechanism of variation potential influence on photosynthesis in pea. Plant Cell Environ..

[29] Sukhov V., Surova L., Sherstneva O., Katicheva L., Vodeneev V. (2015). Variation potential influence on photosynthetic cyclic electron flow in pea. Front. Plant Sci..

[30] Sukhova E., Mudrilov M., Vodeneev V., Sukhov V. (2018). Influence of the variation potential on photosynthetic flows of light energy and electrons in pea. Photosynth. Res..

[31] Sukhov V., Sukhova E., Gromova E., Surova L., Nerush V., Vodeneev V. (2019). The electrical signal-induced systemic photosynthetic response is accompanied by changes in the photochemical reflectance index in pea. Func. Plant Biol..

[32] Yudina L., Sukhova E., Sherstneva O., Grinberg M., Ladeynova M., Vodeneev V., Sukhov V. (2020). Exogenous abscisic acid can influence photosynthetic processes in peas through a decrease in activity of H^+^-ATP-ase in the plasma membrane. Biology.

[33] Furch A.C., van Bel A.J., Fricker M.D., Felle H.H., Fuchs M., Hafke J.B. (2009). Sieve element Ca^2+^ channels as relay stations between remote stimuli and sieve tube occlusion in *Vicia faba*. Plant Cell..

[34] Furch A.C., Zimmermann M.R., Will T., Hafke J.B., van Bel A.J. (2010). Remote-controlled stop of phloem mass flow by biphasic occlusion in *Cucurbita maxima*. J. Exp. Bot..

[35] Shiina T., Tazawa M. (1986). Action potential in *Luffa cylindrica* and its effects on elongation growth. Plant Cell Physiol..

[36] Lautner S., Grams T.E., Matyssek R., Fromm J. (2005). Characteristics of electrical signals in poplar and responses in photosynthesis. Plant Physiol..

[37] Retivin V.G., Opritov V.A., Fedulina S.B. (1997). Generation of action potential induces preadaptation of Cucurbita pepo L. stem tissues to freezing injury. Russ. J. Plant Physiol..

[38] Retivin V.G., Opritov V.A., Lobov S.A., Tarakanov S.A., Khudyakov V.A. (1999). Changes in the resistance of photosynthesizing cotyledon cells of pumpkin seedlings to cooling and heating, as induced by the stimulation of the root system with KCl solution. Russ. J. Plant Physiol..

[39] Sukhov V., Surova L., Sherstneva O., Vodeneev V. (2014). Influence of variation potential on resistance of the photosynthetic machinery to heating in pea. Physiol. Plant..

[40] Sukhov V., Surova L., Sherstneva O., Bushueva A., Vodeneev V. (2015). Variation potential induces decreased PSI damage and increased PSII damage under high external temperatures in pea. Funct. Plant. Biol..

[41] Surova L., Sherstneva O., Vodeneev V., Sukhov V. (2016). Variation potential propagation decreases heat-related damage of pea photosystem I by 2 different pathways. Plant Sign. Behav..

[42] Sukhov V., Gaspirovich V., Mysyagin S., Vodeneev V. (2017). High-temperature tolerance of photosynthesis can be linked to local electrical responses in leaves of pea. Front. Physiol..

[43] Suzuki N., Miller G., Salazar C., Mondal H.A., Shulaev E., Cortes D.F., Shuman J.L., Luo X., Shah J., Schlauch K. (2013). Temporal-spatial interaction between reactive oxygen species and abscisic acid regulates rapid systemic acclimation in plants. Plant Cell..

[44] Stahlberg R., Cosgrove D.J. (1997). The propagation of slow wave potentials in pea epicotyls. Plant Physiol..

[45] Malone M. (1994). Wound-induced hydraulic signals and stimulus transmission in *Mimosa pudica* L.. New Phytol..

[46] Mancuso S. (1999). Hydraulic and electrical transmission of wound-induced signals in *Vitis vinifera*. Aust. J. Plant Physiol..

[47] Stahlberg R., Cleland R.E., van Volkenburgh E., Baluška F., Mancuso S., Volkmann D. (2006). Slow wave potentials—A propagating electrical signal unique to higher plants. Communication in Plants. Neuronal Aspects of Plant Life.

[48] Davies E., Stankovic B., Baluška F., Mancuso S., Volkmann D. (2006). Electrical signals, the cytoskeleton, and gene expression: A hypothesis on the coherence of the cellular responses to environmental insult. Communication in Plants. Neuronal Aspects of Plant Life.

[49] Gindl W., Loppert H.G., Wimmer R. (1999). Relationship between streaming potential and sap velocity in *Salix Alba* L.. Phyton-Ann. Rei. Botanicae..

[50] Gibert D., Le Mouël J.-L., Lambs L., Nicollin F., Perrier F. (2006). Sap flow and daily electric potential variations in a tree trunk. Plant Sci..

[51] Hao Z., Li W., Hao X. (2020). Variations of electric potential in the xylem of tree trunks associated with water content rhythms. J. Exp. Bot..

[52] Huber A.E., Bauerle T.L. (2016). Long-distance plant signaling pathways in response to multiple stressors: The gap in knowledge. J. Exp. Bot..

[53] Farmer E.E., Ryan C.A. (1990). Interplant communication—Airborne methyl jasmonate induces synthesis of proteinase inhibitors in plant leaves. Proc. Natl. Acad. Sci. USA.

[54] Pearce G., Strydom D., Johnson S., Ryan C.A. (1991). A polypeptide from tomato leaves induces wound-inducible proteinase inhibitor proteins. Science.

[55] Peña-Cortés H., Fisahn J., Willmitzer L. (1995). Signals involved in wound-induced proteinase inhibitor II gene expression in tomato and potato plants. Proc. Natl. Acad. Sci. USA.

[56] Miller G., Schlauch K., Tam R., Cortes D., Torres M.A., Shulaev V., Dangl J.L., Mittler R. (2009). The plant NADPH oxidase RBOHD mediates rapid systemic signaling in response to diverse stimuli. Sci. Signal..

[57] Hlavinka J., Nožková-Hlaváčková V., Floková K., Novák O., Nauš J. (2012). Jasmonic acid accumulation and systemic photosynthetic and electrical changes in locally burned wild type tomato, ABA-deficient sitiens mutants and sitiens pre-treated by ABA. Plant Physiol. Biochem..

[58] Choi W.G., Miller G., Wallace I., Harper J., Mittler R., Gilroy S. (2017). Orchestrating rapid long-distance signaling in plants with Ca2+, ROS and electrical signals. Plant J..

[59] Toyota M., Spencer D., Sawai-Toyota S., Jiaqi W., Zhang T., Koo A.J., Howe G.A., Gilroy S. (2018). Glutamate triggers long-distance, calcium-based plant defense signaling. Science.

[60] Malone M. (1992). Kinetics of wound-induced hydraulic signals and variation potentials in wheat seedlings. Planta.

[61] Mancuso S., Mugnai S., Baluška F., Mancuso S., Volkmann D. (2006). Long-distance signal transmission in trees. Communication in Plants. Neuronal Aspects of Plant Life.

[62] Vodeneev V., Orlova A., Morozova E., Orlova L., Akinchits E., Orlova O., Sukhov V. (2012). The mechanism of propagation of variation potentials in wheat leaves. J. Plant Physiol..

[63] Vodeneev V., Mudrilov M., Akinchits E., Balalaeva I., Sukhov V. (2018). Parameters of electrical signals and photosynthetic responses induced by them in pea seedlings depend on the nature of stimulus. Funct. Plant Biol..

[64] Sukhov V., Akinchits E., Katicheva L., Vodeneev V. (2013). Simulation of variation potential in higher plant cells. J. Membr. Biol..

[65] Ladeynova M., Mudrilov M., Berezina E., Kior D., Grinberg M., Brilkina A., Sukhov V., Vodeneev V. (2020). Spatial and temporal dynamics of electrical and photosynthetic activity and the content of phytohormones induced by local stimulation of pea plants. Plants.

[66] Ksenzhek O.S., Volkov A.G. (1998). Plant Energetics.

[67] Rosell J.A., Olson M.E., Anfodillo T. (2017). Scaling of xylem vessel diameter with plant size: Causes, predictions, and outstanding questions. Curr. Forestry Rep..

[68] King A.L. (1947). On a generalization of the Poiseuille Law. Am. J. Phys..

[69] Grinberg M.A., Gudkov S.V., Balalaeva I.V., Gromova E., Sinitsyna Y., Sukhov V., Vodeneev V. (2021). Effect of chronic β-radiation on long-distance electrical signals in wheat and their role in adaptation to heat stress. Environ. Exp. Bot..

[70] Evans M.J., Morris R.J. (2017). Chemical agents transported by xylem mass flow propagate variation potentials. Plant J..

[71] Blyth M.G., Morris R.J. (2019). Shear-enhanced dispersion of a wound substance as a candidate mechanism for variation potential transmission. Front. Plant Sci..

[72] Sachs T. (1972). The Induction of fibre dierentiation in peas. Ann. Bot..

[73] Aloni R., Gad A.E. (1982). Anatomy of the primary phloem fiber system in Pisum sativum. Am. J. Bot..

[74] Belardinelli E., Cavalcanti S. (1992). Theoretical analysis of pressure pulse propagation in arterial vessels. J. Biomech..

[75] Secomb T.W. (2016). Hemodynamics. Compr. Physiol..

[76] Hepworth D.G., Vincent J.F.V. (1998). The mechanical properties of xylem tissue from tobacco plants (*Nicotiana tabacum* ’Samsun’). Ann. Bot..

[77] Gradmann D. (2001). Models for oscillations in plants. Aust. J. Plant Physiol..

